# ChatGPT outperforms crowd workers for text-annotation tasks

**DOI:** 10.1073/pnas.2305016120

**Published:** 2023-07-18

**Authors:** Fabrizio Gilardi, Meysam Alizadeh, Maël Kubli

**Affiliations:** ^a^Department of Political Science, University of Zurich, Zurich 8050, Switzerland

**Keywords:** ChatGPT, text classification, large language models, human annotations, text as data

## Abstract

Many NLP applications require manual text annotations for a variety of tasks, notably to train classifiers or evaluate the performance of unsupervised models. Depending on the size and degree of complexity, the tasks may be conducted by crowd workers on platforms such as MTurk as well as trained annotators, such as research assistants. Using four samples of tweets and news articles (*n* = 6,183), we show that ChatGPT outperforms crowd workers for several annotation tasks, including relevance, stance, topics, and frame detection. Across the four datasets, the zero-shot accuracy of ChatGPT exceeds that of crowd workers by about 25 percentage points on average, while ChatGPT’s intercoder agreement exceeds that of both crowd workers and trained annotators for all tasks. Moreover, the per-annotation cost of ChatGPT is less than $0.003—about thirty times cheaper than MTurk. These results demonstrate the potential of large language models to drastically increase the efficiency of text classification.

Many NLP applications require high-quality labeled data, notably to train classifiers or evaluate the performance of unsupervised models. For example, researchers often aim to filter noisy social media data for relevance, assign texts to different topics or conceptual categories, or measure their sentiment or stance. Regardless of the specific approach used for these tasks (supervised, semisupervised, or unsupervised), labeled data are needed to build a training set or a gold standard against which performance can be assessed. Such data may be available for high-level tasks such as semantic evaluation (1). More typically, however, researchers have to conduct original annotations to ensure that the labels match their conceptual categories (2). Until recently, two main strategies were available. First, researchers can recruit and train coders, such as research assistants. Second, they can rely on crowd workers on platforms such as Amazon Mechanical Turk (MTurk). Often, these two strategies are used in combination: trained annotators create a relatively small gold standard dataset, and crowd workers are employed to increase the volume of labeled data. Trained annotators tend to produce high-quality data but involve significant costs. Crowd workers are a much cheaper and more flexible option, but the quality may be insufficient, particularly for complex tasks and languages other than English. Moreover, there have been concerns that MTurk data quality has decreased (3), while alternative platforms such as CrowdFlower and Figure Eight are no longer practicable options for academic research since they were acquired by Appen, a company that is focused on a business market.

This paper explores the potential of large language models (LLMs) for text-annotation tasks, with a focus on ChatGPT, which was released in November 2022. It demonstrates that zero-shot ChatGPT classifications (that is, without any additional training) outperform MTurk annotations at a fraction of the cost. LLMs have been shown to perform very well for a wide range of purposes, including ideological scaling (4), the classification of legislative proposals (5), the resolution of cognitive psychology tasks (6), and the simulation of human samples for survey research (7). While a few studies suggested that ChatGPT might perform text-annotation tasks of the kinds we have described (8, 9), our work provides a systematic evaluation. Our analysis relies on a sample of 6,183 documents, including tweets and news articles that we collected for a previous study (10) as well as a new sample of tweets posted in 2023. In our previous study, the texts were labeled by trained annotators (research assistants) for five different tasks: relevance, stance, topics, and two kinds of frame detection. Using the same codebooks that we developed to instruct our research assistants, we submitted the tasks to ChatGPT as zero-shot classifications, as well as to crowd workers on MTurk. We then evaluated the performance of ChatGPT against two benchmarks: i) its accuracy, relative to that of crowd workers, and ii) its intercoder agreement, relative to that of crowd workers as well as of our trained annotators. We find that across the four datasets, ChatGPT’s zero-shot accuracy is higher than that of MTurk for most tasks. For all tasks, ChatGPT’s intercoder agreement exceeds that of both MTurk and trained annotators. Moreover, ChatGPT is significantly cheaper than MTurk. ChatGPT’s per-annotation cost is about $0.003 or a third of a cent—about thirty times cheaper than MTurk, with higher quality. At this cost, it might potentially be possible to annotate entire samples or to create large training sets for supervised learning. While further research is needed to better understand how ChatGPT and other LLMs perform in a broader range of contexts, these results demonstrate their potential to transform how researchers conduct data annotations and to disrupt parts of the business model of platforms such as MTurk.

## Results

We use four datasets (*n* = 6,183) including tweets and news articles that we collected and annotated manually for a previous study on the discourse around content moderation (10), as well as a new sample of tweets posted in 2023 to address the concern that ChatGPT might be relying on memorization for texts potentially included in the model’s training dataset. We relied on trained annotators (research assistants) to construct a gold standard for six conceptual categories: relevance of tweets for the content moderation issue (relevant/irrelevant); relevance of tweets for political issues (relevant/irrelevant); stance regarding Section 230, a key part of US internet legislation (keep/repeal/neutral); topic identification (six classes); a first set of frames (content moderation as a problem, as a solution, or neutral); and a second set of frames (fourteen classes). We then performed these exact same classifications with ChatGPT and with crowd workers recruited on MTurk, using the same codebook we developed for our research assistants (*SI Appendix*, S1). For ChatGPT, we conducted four sets of annotations. To explore the effect of ChatGPT’s temperature parameter, which controls the degree of randomness of the output, we conducted the annotations with the default value of 1 as well as with a value of 0.2, which implies less randomness. For each temperature value, we conducted two sets of annotations to compute ChatGPT’s intercoder agreement. For MTurk, we aimed to select high-quality crowd workers, notably by filtering for workers who are classified as “MTurk Masters” by Amazon, who have an approval rate of over 90%, and who are located in the United States. Our procedures are described more in detail in *Materials and Methods*.

**Fig. 1.**
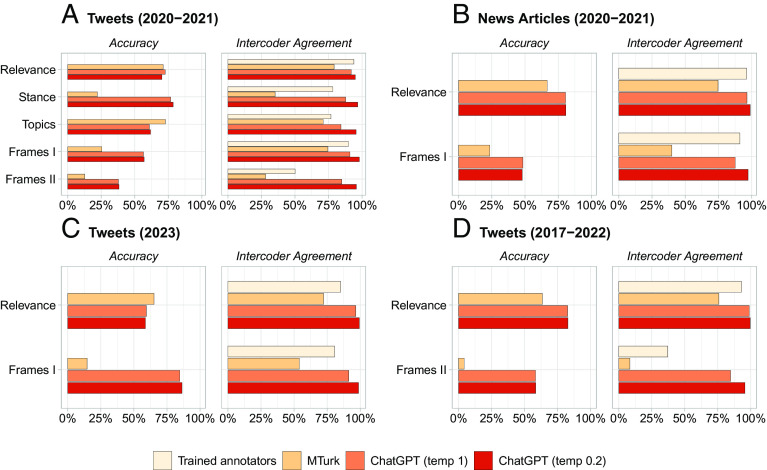
ChatGPT zero-shot text annotation performance in four datasets (A: tweets, 2020-2021; B: news articles, 2020-2021; C: tweets, 2023; D: tweets, 2017-2022), compared to MTurk and trained annotators. ChatGPT’s accuracy outperforms that of MTurk for most tasks. ChatGPT’s intercoder agreement outperforms that of both MTurk and trained annotators in all tasks. Accuracy means agreement with the trained annotators.

Across the four datasets, we report ChatGPT’s zero-shot performance for two different metrics: accuracy and intercoder agreement (Fig. 1). Accuracy is measured as the percentage of correct annotations (using our trained annotators as a benchmark), while intercoder agreement is computed as the percentage of tweets that were assigned the same label by two different annotators (research assistant, crowd workers, or ChatGPT runs). Regarding accuracy, Fig. 1 shows that ChatGPT outperforms MTurk for most tasks across the four datasets. On average, ChatGPT’s accuracy exceeds that of MTurk by about 25 percentage points. Moreover, ChatGPT demonstrates adequate accuracy overall, considering the challenging tasks, number of classes, and zero-shot annotations. Accuracy rates for relevance tasks, with two classes (relevant/irrelevant) are 70% for content moderation tweets, 81% for content moderation news articles, 83% for US Congress tweets, and 59% for 2023 content moderation tweets. In the 2023 sample, ChatGPT performed much better than MTurk in the second task but struggled with misclassifying tweets about specific user suspensions in the relevance task due to a lack of examples in the prompt. While these findings do not suggest that memorization is a major issue, they underscore the importance of high-quality prompts.

Regarding intercoder agreement, Fig. 1 shows that ChatGPT’s performance is very high. On average, intercoder agreement is about 56% for MTurk, 79% for trained annotators, 91% for ChatGPT with temperature = 1, and 97% for ChatGPT with temperature = 0.2. The correlation between intercoder agreement and accuracy is positive (Pearson’s r = 0.36). This suggests that a lower temperature value may be preferable for annotation tasks, as it seems to increase consistency without decreasing accuracy.

We underscore that the test to which we subjected ChatGPT is hard. Our tasks were originally conducted in the context of a previous study (10) and required considerable resources. We developed most of the conceptual categories for our particular research purposes. Moreover, some of the tasks involve a large number of classes and exhibit lower levels of intercoder agreement, which indicates a higher degree of annotation difficulty (11). ChatGPT’s accuracy is positively correlated with the intercoder agreement of trained annotators (Pearson’s r = 0.46), suggesting better performance for easier tasks. Conversely, ChatGPT’s outperformance of MTurk is negatively correlated with the intercoder agreement of trained annotators (Pearson’s r = -0.37), potentially indicating stronger overperformance for more complex tasks.

We conclude that ChatGPT’s performance is impressive, particularly considering that its annotations are zero-shot.

## Discussion

This paper demonstrates the potential of LLMs to transform text-annotation procedures for a variety of tasks common to many research projects. The evidence is consistent across different types of texts and time periods. It strongly suggests that ChatGPT may already be a superior approach compared to crowd annotations on platforms such as MTurk. At the very least, the findings demonstrate the importance of studying the text-annotation properties and capabilities of LLMs more in depth. The following questions seem particularly promising: i) performance across multiple languages; ii) implementation of few-shot learning; iii) construction of semiautomated data labeling systems in which a model learns from human annotations and then recommends labeling procedures (12); iv) using chain of thought prompting and other strategies to increase the performance of zero-shot reasoning (13); and v) comparison across different types of LLMs.

## Materials and Methods

### Datasets.

The analysis relies on four datasets: i) a random sample of 2,382 tweets drawn from a dataset of 2.6 million tweets on content moderation posted from January 2020 to April 2021; ii) a random sample of 1,856 tweets posted by members of the US Congress from 2017 to 2022, drawn from a dataset of 20 million tweets; iii) a random sample of 1,606 articles newspaper articles on content moderation published from January 2020 to April 2021, drawn from a dataset of 980k articles collected via LexisNexis. The sample size was determined by the number of texts needed to build a training set for a machine learning classifier. The fourth dataset iv) replicated the data collection for (i), but for January 2023. It includes a random sample of 500 tweets (of which 339 were in English) drawn from a dataset of 1.3 million tweets.

### Annotation Tasks.

We implemented several annotation tasks: 1) relevance: whether a tweet is about content moderation or, in a separate task, about politics; 2) topic detection: whether a tweet is about a set of six predefined topics (i.e., Section 230, Trump Ban, Complaint, Platform Policies, Twitter Support, and others); 3) stance detection: whether a tweet is in favor of, against, or neutral about repealing Section 230 (a piece of US legislation central to content moderation); 4) general frame detection: whether a tweet contains a set of two opposing frames (“problem” and “solution”). The solution frame describes tweets framing content moderation as a solution to other issues (e.g., hate speech). The problem frame describes tweets framing content moderation as a problem on its own as well as to other issues (e.g., free speech); 5) policy frame detection: whether a tweet contains a set of fourteen policy frames proposed (14). The full text of instruction for the five annotation tasks is presented in *SI Appendix*, S1. We used the exact same wordings for ChatGPT and MTurk.

### Trained Annotators.

We trained three political science students to conduct the annotation tasks. For each task, they were given the same set of instructions described above and detailed in *SI Appendix*, S1. The coders annotated the tweets independently task by task.

### Crowd Workers.

We employed MTurk workers to perform the same set of tasks as trained annotators and ChatGPT, using the same set of instructions (*SI Appendix*, S1). To ensure annotation quality, we restricted access to the tasks to workers who are classified as “MTurk Masters” by Amazon, who have an HIT (Human Intelligence Task) approval rate greater than 90% with at least 50 approved HITs, and who are located in the United States. Moreover, we ensured that no worker could annotate more than 20% of the tweets for a given task. As with the trained human annotators, each tweet was annotated by two different crowd workers.

### ChatGPT.

We used the ChatGPT API with the “gpt-3.5-turbo”. The annotations were conducted between March 9–20 and April 27–May 4, 2023. For each task, we prompted ChatGPT with the corresponding annotation instruction text (*SI Appendix*, S1). We intentionally avoided adding any ChatGPT-specific prompts to ensure comparability between ChatGPT and MTurk crowd workers. After testing several variations, we decided to feed tweets one by one to ChatGPT using the following prompt: “Here’s the tweet I picked, please label it as [Task Specific Instruction (e.g., ‘one of the topics in the instruction’)].” We set the temperature parameter at 1 (default value) and 0.2 (which makes the output more deterministic; higher values make the output more random). For each temperature setting, we collected two responses from ChatGPT to compute the intercoder agreement. That is, we collected four ChatGPT responses for each tweet. We created a new chat session for every tweet to ensure that the ChatGPT results are not influenced by the history of annotations.

### Evaluation Metrics.

First, we computed average accuracy (i.e., percentage of correct predictions), that is, the number of correctly classified instances over the total number of cases to be classified, using trained human annotations as our gold standard and considering only texts that both annotators agreed upon. Second, intercoder agreement refers to the percentage of instances for which both annotators in a given group report the same class.

## Supplementary Material

Appendix 01 (PDF)

## Data Availability

Replication materials are available at the Harvard Dataverse, https://doi.org/10.7910/DVN/PQYF6M (15). Some study data are available (only tweet IDs can be shared, not tweets themselves).
