# Supplementary material for: ChatGPT outperforms crowd workers for text-annotation tasks
Source: Proc Natl Acad Sci U S A. 2023 Jul 18;120(30):e2305016120. doi: 10.1073/pnas.2305016120 (PMC10372638; doi:10.1073/pnas.2305016120)
Supplement: Supplementary file 1 — Appendix 01 (PDF) [file pnas.2305016120.sapp.pdf]

S1. Annotation Codebooks

Not all of the annotations described in these codebooks were conducted for every dataset in our study. First, the manual annotations we use as a benchmark were performed in a previous study, except for the new 2023 sample, which was specifically annotated for this current study. Second, certain annotation tasks are not applicable to all datasets. For instance, stance analysis, problem/solution, and topic modeling were not suitable for analyzing tweets from US Congress members. This is because these tweets cover a wide range of issues and topics, unlike content moderation topics, which are more focused. For news articles, our attempts at human annotation for stance, topic, and policy frames were not successful. This was because the articles primarily revolved around platform policies, actions, and criticisms thereof.

A. Background on content moderation (to be used for all tasks except the tweets from US Congressmembers). For this task, you will be asked to annotate a sample of tweets about content moderation. Before describing the task, we explain what we mean by "content moderation".

"Content moderation" refers to the practice of screening and monitoring content posted by users on social media sites to determine if the content should be published or not, based on specific rules and guidelines. Every time someone posts something on a platform like Facebook or Twitter, that piece of content goes through a review process ("content moderation") to ensure that it is not illegal, hateful or inappropriate and that it complies with the rules of the site. When that is not the case, that piece of content can be removed, flagged, labeled as or 'disputed.'

Deciding what should be allowed on social media is not always easy. For example, many sites ban child pornography and terrorist content as it is illegal. However, things are less clear when it comes to content about the safety of vaccines or politics, for example. Even when people agree that some content should be blocked, they do not always agree about the best way to do so, how effective it is, and who should do it (the government or private companies, human moderators, or artificial intelligence).

B. Background on political tweets (to be used for tweets by the US Congress members). For this task, you will be asked to annotate a sample of tweets to determine if they include political content or not. For the purposes of this task, tweets are "relevant" if they include political content, and "irrelevant" if they do not. Before describing the task, we explain what we mean by "political content".

"Political content" refers to any tweets that pertain to politics or government policies at the local, national, or international level. This can include tweets that discuss political figures, events, or issues, as well as tweets that use political language or hashtags. To determine if tweets include political content or not, consider several factors, such as the use of political keywords or hashtags, the mention of political figures or events, the inclusion of links to news articles or other political sources, and the overall tone and sentiment of the tweet, which may indicate whether it is conveying a political message or viewpoint.

C. Task 1: Relevance (Content Moderation). For each tweet in the sample, follow these instructions:

1. Carefully read the text of the tweet, paying close attention to details.
2. Classify the tweet as either relevant (1) or irrelevant (0)

Tweets should be coded as RELEVANT when they directly relate to content moderation, as defined above. This includes tweets that discuss: social media platforms' content moderation rules and practices, governments' regulation of online content moderation, and/or mild forms of content moderation like flagging.

Tweets should be coded as IRRELEVANT if they do not refer to content moderation, as defined above, or if they are themselves examples of moderated content. This would include, for example, a Tweet by Donald Trump that Twitter has labeled as "disputed", a tweet claiming that something is false, or a tweet containing sensitive content. Such tweets might be subject to content moderation, but are not discussing content moderation. Therefore, they should be coded as irrelevant for our purposes.

D. Task 2: Relevance (Political Content). For each tweet in the sample, follow these instructions:

1. Carefully read the text of the tweet, paying close attention to details.
2. Classify the tweet as either relevant (1) or irrelevant (0)

Tweets should be coded as RELEVANT if they include POLITICAL CONTENT, as defined above. Tweets should be coded as IRRELEVANT if they do NOT include POLITICAL CONTENT, as defined above.

E. Task 3: Problem/Solution Frames. Content moderation can be seen from two different perspectives:

- Content moderation can be seen as a PROBLEM; for example, as a restriction of free speech
- Content moderation can be seen as a SOLUTION; for example, as a protection from harmful speech

For each tweet in the sample, follow these instructions:

1. Carefully read the text of the tweet, paying close attention to details.
2. Classify the tweet as describing content moderation as a problem, as a solution, or neither.

Tweets should be classified as describing content moderation as a PROBLEM if they emphasize negative effects of content moderation, such as restrictions to free speech, or the biases that can emerge from decisions regarding what users are allowed to post.

Tweets should be classified as describing content moderation as a SOLUTION if they emphasize positive effects of content moderation, such as protecting users from various kinds of harmful content, including hate speech, misinformation, illegal adult content, or spam.

Tweets should be classified as describing content moderation as NEUTRAL if they do not emphasize possible negative or positive effects of content moderation, for example if they simply report on the content moderation activity of social media platforms without linking them to potential advantages or disadvantages for users or stakeholders.

F. Task 4: Policy Frames (Content Moderation). Content moderation, as described above, can be linked to various other topics, such as health, crime, or equality.

For each tweet in the sample, follow these instructions:

1. Carefully read the text of the tweet, paying close attention to details.
2. Classify the tweet into one of the topics defined below.

The topics are defined as follows:

- ECONOMY: The costs, benefits, or monetary/financial implications of the issue (to an individual, family, community, or to the economy as a whole).
- Capacity and resources: The lack of or availability of physical, geographical, spatial, human, and financial resources, or the capacity of existing systems and resources to implement or carry out policy goals.
- MORALITY: Any perspective—or policy objective or action (including proposed action)that is compelled by religious doctrine or interpretation, duty, honor, righteousness or any other sense of ethics or social responsibility.
- FAIRNESS AND EQUALITY: Equality or inequality with which laws, punishment, rewards, and resources are applied or distributed among individuals or groups. Also the balance between the rights or interests of one individual or group compared to another individual or group.
- CONSTITUTIONALITY AND JURISPRUDENCE: The constraints imposed on or freedoms granted to individuals, government, and corporations via the Constitution, Bill of Rights and other amendments, or judicial interpretation. This deals specifically with the authority of government to regulate, and the authority of individuals/corporations to act independently of government.

DRAFT

- POLICY PRESCRIPTION AND EVALUATION: Particular policies proposed for addressing an identified problem, and figuring out if certain policies will work, or if existing policies are effective.
- LAW AND ORDER, CRIME AND JUSTICE: Specific policies in practice and their enforcement, incentives, and implications. Includes stories about enforcement and interpretation of laws by individuals and law enforcement, breaking laws, loopholes, fines, sentencing and punishment. Increases or reductions in crime.
- SECURITY AND DEFENSE: Security, threats to security, and protection of one's person, family, in-group, nation, etc. Generally an action or a call to action that can be taken to protect the welfare of a person, group, nation sometimes from a not yet manifested threat.
- HEALTH AND SAFETY: Health care access and effectiveness, illness, disease, sanitation, obesity, mental health effects, prevention of or perpetuation of gun violence, infrastructure and building safety.
- QUALITY OF LIFE: The effects of a policy on individuals' wealth, mobility, access to resources, happiness, social structures, ease of day-to-day routines, quality of community life, etc.
- CULTURAL IDENTITY: The social norms, trends, values and customs constituting culture(s), as they relate to a specific policy issue.
- PUBLIC OPINION: References to general social attitudes, polling and demographic information, as well as implied or actual consequences of diverging from or "getting ahead of" public opinion or polls.
- POLITICAL: Any political considerations surrounding an issue. Issue actions or efforts or stances that are political, such as partisan filibusters, lobbyist involvement, bipartisan efforts, deal-making and vote trading, appealing to one's base, mentions of political maneuvering. Explicit statements that a policy issue is good or bad for a particular political party.
- EXTERNAL REGULATION AND REPUTATION: The United States' external relations with another nation; the external relations of one state with another; or relations between groups. This includes trade agreements and outcomes, comparisons of policy outcomes or desired policy outcomes.
- OTHER: Any topic that does not fit into the above categories.

G. Task 5: Policy Frames (Political Content). Political content, as described above, can be linked to various other topics, such as health, crime, or equality.

For each tweet in the sample, follow these instructions:

1. Carefully read the text of the tweet, paying close attention to details.
2. Classify the tweet into one of the topics defined below.

The topics are defined as follows:

- ECONOMY: The costs, benefits, or monetary/financial implications of the issue (to an individual, family, community, or to the economy as a whole).
- Capacity and resources: The lack of or availability of physical, geographical, spatial, human, and financial resources, or the capacity of existing systems and resources to implement or carry out policy goals.
- MORALITY: Any perspective—or policy objective or action (including proposed action)that is compelled by religious doctrine or interpretation, duty, honor, righteousness or any other sense of ethics or social responsibility.
- FAIRNESS AND EQUALITY: Equality or inequality with which laws, punishment, rewards, and resources are applied or distributed among individuals or groups. Also the balance between the rights or interests of one individual or group compared to another individual or group.
- CONSTITUTIONALITY AND JURISPRUDENCE: The constraints imposed on or freedoms granted to individuals, government, and corporations via the Constitution, Bill of Rights and other amendments, or judicial interpretation. This deals specifically with the authority of government to regulate, and the authority of individuals/corporations to act independently of government.
- POLICY PRESCRIPTION AND EVALUATION: Particular policies proposed for addressing an identified problem, and figuring out if certain policies will work, or if existing policies are effective.
- LAW AND ORDER, CRIME AND JUSTICE: Specific policies in practice and their enforcement, incentives, and implications. Includes stories about enforcement and interpretation of laws by individuals and law enforcement, breaking laws, loopholes, fines, sentencing and punishment. Increases or reductions in crime.
- SECURITY AND DEFENSE: Security, threats to security, and protection of one's person, family, in-group, nation, etc. Generally an action or a call to action that can be taken to protect the welfare of a person, group, nation sometimes from a not yet manifested threat.
- HEALTH AND SAFETY: Health care access and effectiveness, illness, disease, sanitation, obesity, mental health effects, prevention of or perpetuation of gun violence, infrastructure and building safety.
- QUALITY OF LIFE: The effects of a policy on individuals' wealth, mobility, access to resources, happiness, social structures, ease of day-to-day routines, quality of community life, etc.
- CULTURAL IDENTITY: The social norms, trends, values and customs constituting culture(s), as they relate to a specific policy issue.
- PUBLIC OPINION: References to general social attitudes, polling and demographic information, as well as implied or actual consequences of diverging from or "getting ahead of" public opinion or polls.
- POLITICAL: Any political considerations surrounding an issue. Issue actions or efforts or stances that are political, such as partisan filibusters, lobbyist involvement, bipartisan efforts, deal-making and vote trading, appealing to one's base, mentions of political maneuvering. Explicit statements that a policy issue is good or bad for a particular political party.
- EXTERNAL REGULATION AND REPUTATION: The United States' external relations with another nation; the external relations of one state with another; or relations between groups. This includes trade agreements and outcomes, comparisons of policy outcomes or desired policy outcomes.
- OTHER: Any topic that does not fit into the above categories.

DRAFT

H. Task 6: Stance Detection. In the context of content moderation, Section 230 is a law in the United States that protects websites and other online platforms from being held legally responsible for the content posted by their users. This means that if someone posts something illegal or harmful on a website, the website itself cannot be sued for allowing it to be posted. However, websites can still choose to moderate content and remove anything that violates their own policies.

For each tweet in the sample, follow these instructions:

1. Carefully read the text of the tweet, paying close attention to details.
2. Classify the tweet as having a positive stance towards Section 230, a negative stance, or a neutral stance.

I. Task 7: Topic Detection. Tweets about content moderation may also discuss other related topics, such as:

1. Section 230, which is a law in the United States that protects websites and other online platforms from being held legally responsible for the content posted by their users (SECTION 230).

2. The decision by many social media platforms, such as Twitter and Facebook, to suspend Donald Trump's account (TRUMP BAN).
3. Requests directed to Twitter's support account or help center (TWITTER SUPPORT).
4. Social media platforms' policies and practices, such as community guidelines or terms of service (PLATFORM POLICIES).
5. Complaints about platform's policy and practices in deplatforming and content moderation or suggestions to suspend particular accounts, or complaints about accounts being suspended or reported (COMPLAINTS).
6. If a text is not about the SECTION 230, COMPLAINTS, TRUMP BAN, TWITTER SUPPORT, and PLATFORM POLICIES, then it should be classified in OTHER class (OTHER).

For each tweet in the sample, follow these instructions:

1. Carefully read the text of the tweet, paying close attention to details.
2. Please classify the following text according to topic (defined by function of the text, author's purpose and form of the text). You can choose from the following classes: SECTION 230, TRUMP BAN, COMPLAINTS, TWITTER SUPPORT, PLATFORM POLICIES, and OTHER

DRAFT
